# Bone turnover markers in the management of CKD-associated osteoporosis—a European consensus

**DOI:** 10.1093/ndt/gfag033

**Published:** 2026-02-17

**Authors:** Mathias Haarhaus, Hanne Skou Jørgensen, Justine Bacchetta, Anibal Ferreira, Serge Ferrari, Maria Fusaro, Ditte Hansen, Markus Ketteler, Marie-Hélène Lafage-Proust, Alexander D Lalayiannis, Per Magnusson, Sandro Mazzaferro, Syazrah Salam, Rukshana Shroff, Etienne Cavalier, Richard Eastell, Pieter Evenepoel

**Affiliations:** Division of Renal Medicine, Department of Clinical Science, Intervention and Technology, Karolinska Institutet, Karolinska Universitetssjukhuset, Stockholm, Sweden; Diaverum AB, Malmö, Sweden; Department of Renal Medicine, Aarhus University Hospital, Aarhus, Denmark; Department of Clinical Medicine, Aarhus University, Aarhus, Denmark; Reference Center for Rare Diseases of Calcium and Phosphate, INSERM1033 Research Unit, Université Lyon, Lyon, France; Department of Pediatric Nephrology, Hospices Civils de Lyon, Lyon, France; Nova Medical School, Lisbon, Portugal; Department of Nephrology, Hospital Curry Cabral, Lisbon, Portugal; Geneva University Hospital, Geneva, Switzerland; National Research Council, Institute of Clinical Physiology, Pisa, Italy; Department of Medicine, University of Padua, Padua, Italy; Department of Clinical Medicine, University of Copenhagen, Copenhagen, Denmark; Department of Nephrology, Copenhagen University Hospital – Herlev, Herlev, Denmark; Department of General Internal Medicine and Nephrology, Robert-Bosch-Hospital, Stuttgart, Germany; INSERM U1059 SAINBIOSE Université Jean Monnet, Mines Saint-Etienne, Saint-Etienne, France; Department of Rheumatology, CHU, Saint-Priest-en-Jarez, France; Department of Pediatric Nephrology, Birmingham Women’s and Children’s NHS Foundation Trust, Birmingham, UK; Department of Clinical Chemistry, and Department of Biomedical and Clinical Sciences, Linköping University, Linköping, Sweden; Department of Translation and Precision Medicine, Sapienza University of Rome, Roma, Italy; Division of Clinical Medicine, School of Medicine and Population Health, University of Sheffield, Sheffield, UK; Metabolic Bone Centre and Sheffield Kidney Institute, Sheffield Teaching Hospitals NHS Foundation Trust, Sheffield, UK; University College London, Institute of Child Health, London, UK; UCL Great Ormond Street Hospital for Children NHS Foundation Trust, London, UK; Department of Clinical Chemistry, CIRM, University of Liège, CHU de Liège, Liege, Belgium; Division of Clinical Medicine, School of Medicine and Population Health, University of Sheffield, Sheffield, UK; Department of Microbiology, Immunology and Transplantation, Nephrology and Renal Transplantation Research Group, Katholieke Universiteit Leuven, Leuven, Belgium; Department of Medicine, Division of Nephrology, University Hospitals Leuven, Leuven, Belgium

**Keywords:** alkaline phosphatase, bone mineral density, chronic kidney disease - mineral and bone disorder, osteoporosis, renal osteodystrophy

## Abstract

Bone turnover abnormalities are common in chronic kidney disease (CKD) and contribute to bone fragility. Recently, the Kidney Disease: Improving Global Outcomes (KDIGO) introduced the term CKD-associated osteoporosis to describe bone fragility in CKD and highlighted the role of bone turnover markers (BTM) in clinical evaluation and management of bone fragility. However, current clinical practice guidelines lack specific treatment targets for bone turnover in the setting of CKD. The aim of this consensus was to present recommendations for the use of BTM in the management of CKD-associated osteoporosis. The consensus was conducted by members of relevant working groups of the European Renal Association (ERA), International Osteoporosis Foundation (IOF), European Foundation for Clinical Chemistry and Laboratory Medicine (EFLM) and European Society of Pediatric Nephrology (ESPN). Bone-specific alkaline phosphatase and tartrate resistant acid phosphatase isoform 5b are not renally cleared and considered reliable diagnostic tools for bone evaluation in CKD-associated osteoporosis. Both have been proposed as reference BTM in CKD by a recent international consortium. Intact N-terminal telopeptide of type I collagen can also be used in CKD. The current consensus describes methodological considerations and the clinical usefulness of BTM in risk prediction, choice of therapeutic strategy, and treatment evaluation in CKD-associated osteoporosis. Specific diagnostic ranges for bone turnover abnormalities are provided. Future research should focus on the integration of these reference BTM with other diagnostic tools, establishment of concrete treatment targets, and development of treatment strategies to reach these targets.

## Graphical Abstract

**Graphical Abstract:**
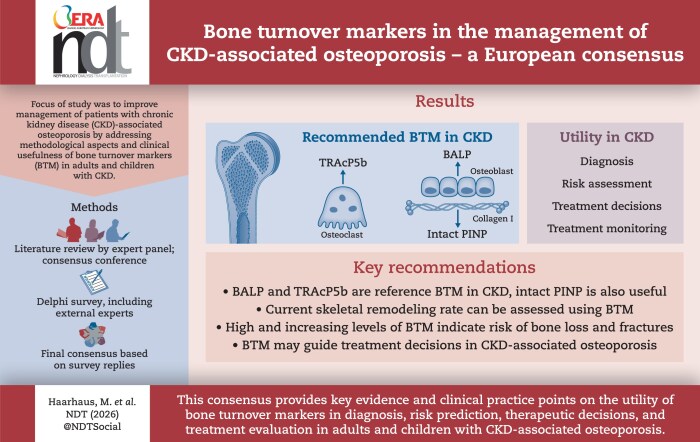

## INTRODUCTION

Bone turnover abnormalities are common in chronic kidney disease (CKD) and contribute to bone fragility [1]; this may explain, at least in part, the exceptionally high risk of fractures in patients with this condition. The current management of mineral and bone disorders in CKD focuses on parathyroid hormone (PTH), vitamin D, calcium and phosphate as risk markers and treatment targets. However, the resulting treatment strategies have not been able to reduce fracture risk substantially [2]. The term CKD-associated osteoporosis was introduced at the 2023 Kidney Disease: Improving Global Outcomes (KDIGO) controversies conference on CKD–mineral and bone disorder (CKD-MBD) to foster a more holistic, patient-centric view on bone fragility in CKD [3]. CKD-associated osteoporosis combines features of renal osteodystrophy, such as abnormalities of bone turnover, mineralization, volume and material properties, with features of primary (postmenopausal or age-related) and drug-induced osteoporosis. Diagnostic work-up and therapeutic options for CKD-associated osteoporosis are largely interchangeable with other osteoporosis conditions, but must be tailored to the specific situation of CKD, with patients often demonstrating more extensive bone turnover abnormalities compared with primary or other secondary forms of osteoporosis. Knowledge of bone turnover may thus be important for the diagnosis and management of CKD-associated osteoporosis since it may identify patients at high fracture risk, assist in determining whether optimization of mineral metabolism disturbances are needed, be supportive in the choice of anti-osteoporotic agent (e.g. anti-resorptive vs anabolic agents) and be useful for the evaluation of treatment effects.

A shift towards a greater consideration of target organ damage and, thus, a more active role of bone turnover markers (BTM) in the management of CKD-associated osteoporosis was advocated by the 2023 KDIGO Controversies Conference [3]. In parallel, an update of the recommendations for reference BTM in the management of osteoporosis suggests, for the first time, bone-specific alkaline phosphatase (BALP) and tartrate-resistant acid phosphatase 5b (TRACP5b) as the reference BTM for patients with CKDG4–5D [3, 4]. Future research in CKD-associated osteoporosis should include these two markers.

The 2017 update of the KDIGO CKD-MBD guidelines suggested using PTH for the evaluation of bone turnover, acknowledging a wide area of uncertainty (target range 2–9 times the upper normal limit of the assay) [5]. This unfortunate characterization of PTH as a BTM has caused some confusion in the Nephrology community. It is increasingly recognized that PTH is a bone regulatory molecule, reflecting parathyroid function, and not a BTM. Analytical heterogeneity and variable skeletal response to PTH further complicates the value of PTH as a BTM in the setting of CKD. The KDIGO guidelines further recommend BALP as an alternative marker for the evaluation of bone turnover, and a bone biopsy if knowledge of the type of renal osteodystrophy will impact treatment decisions. However, bone biopsies are poorly suited for routine evaluation in everyday clinical practice, especially in the setting of longitudinal follow-up. Furthermore, the KDIGO guidelines lack target definitions for BALP and bone turnover.

Thus, clinicians are left with a high degree of uncertainty when initiating and evaluating interventions targeting bone turnover. The aim of this consensus was to present clinicians with an up-to date literature review and recommendations for the use of BTM in the management of CKD-associated osteoporosis, based on current evidence and expert opinion.

## MATERIALS AND METHODS

The current consensus was initiated by the European Renal Osteodystrophy (EUROD) Initiative of the European Renal Association (ERA) CKD-MBD Working Group. Invitations were extended to other organisations with relevance for the management of CKD-associated osteoporosis, i.e. the International Osteoporosis Foundation (IOF), the European Foundation for Clinical Chemistry and Laboratory Medicine (EFLM), and the European Society of Pediatric Nephrology (ESPN). Representatives from each organisation were invited to join the writing team, based on their expertise. Those who consented and contributed are listed as co-authors.

In the run-up to this consensus initiative, a survey on the use of BTM in the setting of CKD was constructed to gain insight into current practice patterns. The survey was circulated among members of the CKD-MBD working group of the ERA. Responses helped select the topics for this consensus initiative. The survey and replies can be found in Supplementary data, Tables S1 and S2.

Writing team members were allocated to one or two topics, with three to four experts per topic, and requested to review and summarise available evidence. Strategies for the literature search are listed in Supplementary data, Table S3. Results of the literature search were discussed by all consensus members at a face-to-face conference. Based on these discussions, ‘key evidence points’, summarising the evidence, and ‘clinical practice points’, presenting actionable recommendations for clinical practice, were constructed. All statements were collected in an e-questionnaire and sent to a panel of experts, consisting of members of all participating societies, in a Delphi method format. The expert panel was asked to provide their agreement with the statements on a 5-point scale (strongly agree, agree, neither agree nor disagree, disagree, strongly disagree) with the opportunity of suggesting rewording of the statements or providing direct feedback. The Delphi survey and its results can be found in Supplementary data, Fig. S1. It was decided *a priori* that at least a 70% level of agreement and a level of disagreement of <5% were required for each statement, failing which the recommendation would be adapted after discussion by the consensus members. The final manuscript was sent to experts from each participating society for review and approval.

## RESULTS

### Methodological aspects of BTM in CKD

*Key evidence points*

- For clinical applicability, circulating BTM are defined as: (i) matrix-derived markers in the form of collagen fragments [C- or N-terminal telopeptide of type I collagen (CTX or NTX) and pro-collagen type I C- or N-terminal propeptides (PICP or PINP)], (ii) enzymes released from bone cells (BALP, TRACP5b).
- The matrix-derived BTM recommended for use in postmenopausal osteoporosis [β-CTX-I and total PINP (tPINP)] accumulate with kidney dysfunction and are thus difficult to interpret in the context of CKD.
- Intact PINP (iPINP), BALP and TRACP5b are minimally affected by kidney function, circadian variation, and feeding.
- Commercially available analytical assays for BTM are hampered by lack of standardisation and harmonisation.

*Clinical practice points*

- BALP and TRACP5b are recommended as reference markers to assess bone turnover (BALP for formation, TRACP5b for resorption) in CKD.
- In addition to the reference markers, iPINP can be used to estimate bone formation in CKD.
- Circulating BTM should be interpreted with knowledge of appropriate reference intervals, preanalytical factors, limitations of the assays, and potential cross-reactivities.
- Longitudinal changes of BTM should make use of the same commercial analytical assay and be interpreted with knowledge of biological variability and least significant change.
- Total ALP may serve as a proxy of BALP in the absence of liver dysfunction, intestinal disease, and inflammation.

### Rationale

**Figure 1:**
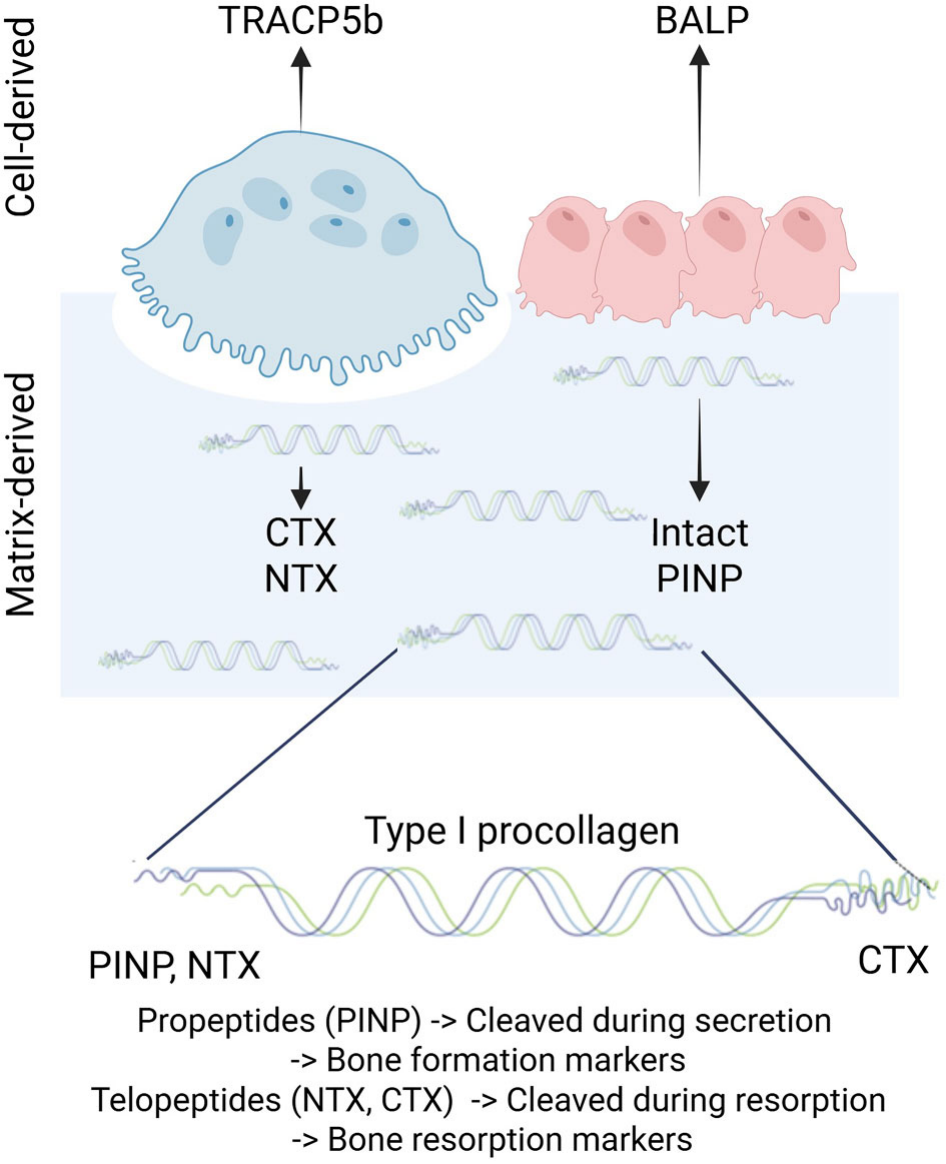
Circulating bone turnover markers are substances released during the process of skeletal remodelling which can be used to monitor these processes in bone. Collagen (C- or N-terminal telopeptide of type I collagen, CTX or NTX) and pro-collagen (pro-collagen type I C- or N-terminal propeptides, PICP or PINP) fragments are passively released during bone resorption or bone formation, respectively, while bone cell enzymes reflect the number or activity of osteoblasts (BALP) and osteoclasts (TRACP5b).

At present, European clinical laboratories are using units, nomenclature, and abbreviations for biochemical indices of bone status at their own discretion, leading to confusion. The scientific literature follows the same path, which complicates comparison of studies and aggregation of data. To overcome this issue, the IOF and the International Federation of Clinical Chemistry (IFCC) joint Committee on Bone Metabolism recently published a position statement on the correct use of nomenclature [6]. According to this work, bone status indices are referred to as (i) matrix-derived BTM in the form of collagen fragments released during formation or resorption of bone tissue, (ii) enzymes released from bone cells or (iii) molecules regulating bone metabolism. Matrix-derived BTM and enzymes released from bone cells inform on bone cell number and/or activity (Fig. 1). For the sake of clinical applicability, they are summarized as circulating BTM in this consensus, as they relate to bone turnover on histomorphometric analysis of bone biopsies (see section **Diagnostic accuracy of BTM** for detailed discussion):

- Tissue-nonspecific alkaline phosphatase (TNSALP) is encoded by the *ALPL* gene. TNSALP goes through post-translational glycosylation, is expressed as BALP in osteoblasts, and is released into the circulation in a manner proportional with the number and differentiation state of osteoblasts [7].
- Osteoblasts also secrete type I collagen as an intact molecule containing the N- and C-terminal propeptides, which are subsequently cleaved in the extracellular space. Thus, PINP and C-terminal propeptides of type I collagen (PICP) levels are markers of type I collagen synthesis by osteoblasts. PINP is the preferred marker, since it has been more extensively studied and since clearance of circulating PICP appears to be under endocrine control [8]. PINP is present initially as an intact trimer (iPINP) of the propeptides from the three protein chains in type I collagen (∼35 kDa) and is subsequently converted to monomeric forms of lower molecular weight in circulation.
- TRACP5 is a metalloenzyme that is synthesised as a highly glycosylated single chain 35 kDa isoform, denoted TRACP5a, with a peptide loop that interacts with the active site restricting the catalytic activity [9]. Proteolytic cleavage by cathepsin K in the loop domain results in a dimer with two subunits of 16 and 23 kDa, *isoform TRACP5b*, with augmented phosphatase activity [10]. The TRACP5b isoform is considered osteoclast-specific while the TRACP5a isoform is a marker of adipose tissue regulation and inflammatory conditions [11]. Circulating TRACP5b reflects the number of osteoclasts, rather than their activity [12].
- C- and N-terminal telopeptides of type I collagen (CTX-I and NTX-I) are both cleaved during osteoclastic resorption of bone, resulting in their liberation into the circulation at a rate proportional to bone resorption activity.

The BTM recommended as reference biomarkers in postmenopausal osteoporosis, β-isomerized CTX-I (β-CTX-I) [13] and tPINP [14] are affected by kidney dysfunction, and therefore less appropriate for use in the setting of CKD. More specifically, degradation products of both proteins are eliminated by the kidneys and will accumulate in the setting of CKD. As a consequence, measurement of β-CTX-I and tPINP, which includes both the trimeric form and monomeric breakdown products, most likely overestimates bone turnover in CKD [15].

Recently, the European Society for Clinical and Economic Aspects of Osteoporosis, Osteoarthritis and Musculoskeletal Diseases (ESCEO) together with the IFCC and the IOF published an update on the role of BTM for the management of osteoporosis. The consensus defined BALP [16] and TRACP5b as reference markers in CKD-associated osteoporosis, reflecting bone formation and resorption, respectively [4]. Assays measuring iPINP may be considered as an alternative or adjunct to BALP as a marker of bone formation.

#### Pre-analytical considerations

Both serum and EDTA plasma matrices can be used for the analysis of PINP (tPINP and iPINP) by radioimmunoassay and automated immunoassays [17]. BALP can be measured in serum or lithium heparin plasma. EDTA leads to irreversible inactivation of ALP activity due to removal of magnesium and essential zinc ions [18]. TRACP5b can be measured in serum and EDTA plasma, but the same specimen type should be used throughout in longitudinal studies and for monitoring clinical patient samples. PINP, BALP and TRACP5b are minimally affected by circadian variation and feeding in contrast to β-CTX-I [19].

#### Analytical considerations

There are several ways to measure BALP, but immunoassays are the most appropriate for use in clinical context, because they are rapid, easy to use, reproducible and demonstrate sufficient specificity in the absence of increased circulating liver isoforms, with which they cross-react [20, 21]. Results are reported in mass (µg/L, Ostase^®^) or activity (U/L, MicroVue™ formerly Alkphase™). Current automatic BALP immunoassays do not measure mass directly. They are based on the original Ostase^®^ assay, which converts the measured enzymatic activity to µg/L by applying a standard curve where the enzymatic BALP activity originally was expressed as mass units (µg/L) based on the estimated molecular mass of 68 kDa (monomeric BALP) [22, 23]. Today, we know that four different BALP isoforms exist, circulating as homodimers, with molecular weights varying from 126 to 141 kDa [24]. Transferring measured BALP activities to mass units appears incongruous and makes it difficult to compare data with activity-based BALP and total ALP assays. All commercially available standards/calibrators used in BALP immunoassays are BALP purified from SaOS-2 cells. SaOS-2 is a heterogeneous osteoblast-like cell line with respect to the expression of BALP. Different companies use different manufacturing processes for purifying BALP from SaOS-2 cells, which results in different enzymatic properties for these standards. The amount of various BALP isoforms differ considerably between the Ostase^®^ and MicroVue™ assays [25]. Taken together, there is a compelling need for harmonisation and standardisation of currently used BALP immunoassays and standards. Immunoassays for the determination of BALP should be interpreted with caution in individuals with known hepatobiliary disease because of cross-reactivity (up to 18%) with the liver ALP isoforms [20]. Bone, liver, and other isoforms of TNSALP only differ by the extent of post-translational glycosylation. Acknowledging that BALP and liver ALP represent the most abundant isoforms in serum, collectively accounting for >90% of total serum ALP [26], total ALP may serve as a proxy of BALP in the absence of liver dysfunction. However, intestinal disease and inflammation may cause increase of other circulating non-skeletal TNSALP isoforms and intestinal ALP, so caution is also warranted when considering total ALP as a proxy of BALP in patients presenting with these conditions [27, 28].

There are four commercially available assays for measuring either total or intact PINP, using various manual and automated methods. Several immunoassays are currently available for measuring PINP in serum, but they vary in their analytical specificity and calibration approaches, which bears significant clinical relevance. True iPINP assays—such as the Orion Diagnostica RIA and IDS iSYS CLIA—specifically detect the trimeric form, while assays labeled ‘total PINP’, including Roche Elecsys ECLIA, detect both the intact trimer and monomeric fragments [14, 29].

In patients with reduced kidney function, corresponding to estimated glomerular filtration rate <30 mL/min/1.73 m², iPINP should be used instead of total PINP [30]. Although results are expressed in µg/L, these immunoassays do not directly measure protein mass. Instead, concentrations are inferred using manufacturer-specific calibrators; Roche uses a synthetic peptide, whereas Orion and IDS rely on purified human trimeric PINP [17]. The absence of a shared calibrator likely contributes to the observed inter-assay variability, particularly between Roche and Orion platforms [17]. However, head-to-head studies report strong concordance between the IDS iSYS and Roche Elecsys assays in patients with normal kidney function, suggesting they may be used interchangeably in this population [31].

It should also be emphasised that PINP clearance is not solely through the kidneys. In patients with alcoholic liver disease, hepatic extraction is markedly impaired, resulting in elevated circulating concentrations of both iPINP and tPINP, despite normal bone turnover [32]. These findings are supported by direct measurements of hepatic and kidney clearance in patients with cirrhosis, showing significantly reduced hepatic extraction compared with controls [33]. Clinicians should therefore avoid interpreting elevated PINP values as evidence of increased bone formation in patients with advanced liver disease, and iPINP should not be used as an alternative to BALP in this context. Ongoing efforts from the IFCC IOF Joint Committee on Bone Metabolism aim to develop a reference measurement procedure and a harmonised calibrator to improve comparability across methods. Until full harmonisation is achieved, clinicians should interpret tPINP results with caution, particularly in patients with CKD or those receiving treatments that may affect kidney clearance of PINP fragments, e.g. non-steroidal anti-inflammatory drugs or aminoglycosides.

Several immunoassays have also been developed that are capable of quantifying TRACP5b and that are insensitive to haemolysis, as opposed to first generation kinetic assays. Automatic analysis of TRACP5b is limited to one company on the IDS iSYS platform, whereas the Nittobo Boseki EIA assay is widely used in Japan. These assays have shown excellent concordance when compared with each other [34].

Lack of harmonisation of analytical methods leads to great confusion in the interpretation of results, especially when derived from different clinical laboratories, or when clinical laboratories switch from one method to another. It is, however, reassuring that available assays for TRACP5b yield similar results [34]. Assays for BALP and iPINP available in Europe have also shown reasonable concordance [35, 36]. Nevertheless, further efforts are needed to improve harmonisation and standardisation of bone status indices, with recent advances in higher order methods like tandem-mass spectrometry creating opportunities [37]. In the position paper recently published by ESCEO/IOF/IFCC on BTM, the authors critically reviewed the existing literature on applicable reference intervals [4]. Their findings highlight a concerning lack of data and scarcity of high-quality studies. Notably, reference values specifically for the CKD population are conspicuously absent. Addressing these gaps should be a priority in the coming years.

In clinical decision-making, it is crucial to know whether a difference observed between two consecutive measurements of a biomarker can be explained by biological and analytical variation. Values of the least significant change (LSC), synonymous with the reference change values (RCV), derive from the biological variability and analytical coefficients of variation (CV) of the method used and provide thresholds to identify changes beyond natural variability. EFLM provides an online calculator to compute the LSC values (alt. RCV) for most biochemical analytes, including those used for monitoring bone turnover, on their website (https://biologicalvariation.eu/). The LSC values for indices of bone status suitable for CKD-associated osteoporosis can be found in Table 1.

**Table 1:** LSC values of the most important biomarkers of bone metabolism used in the follow-up of CKD patients.

Bone status indices	CVA%	CVI%	LSC (% increase)	LSC (% decrease)
PTH	7.4	14.7	46.2	–31.6
BALP	3.3	6.6	18.6	–15.7
iPINP	4.4	8.8	25.6	–20.4
TRACP5b	5.4	10.8	32.2	–24.4

The LSC values have been calculated with a probability of 95%, with a two sides approach (increase and decrease). The desirable CV (half of the CVI) has been used to generate the LSC, but for more precision, the local CVA should be used. CVA, analytical coefficient of variation; CVI, intra-individual variation; RCV, reference change value.

### Diagnostic accuracy of BTM

*Key evidence points*

- Circulating levels of BTM correlate with other measures of skeletal remodelling such as bone histomorphometry and radiotracer kinetics.
- The diagnostic accuracy of BTM depends on the comparator method as well as the pre-test probability of the condition investigated (low or high bone turnover).
- Due to the lack of standardisation, method-specific diagnostic thresholds should be applied.
- BTM show good diagnostic performance for a diagnosis of low or high bone turnover by histomorphometry with area under the curve (AUC) values in the range of 0.70–0.90.

*Clinical practice points*

- A low bone turnover state may be suspected if BALP is <20 µg/L (IDS iSYS) or 30 U/L (Quidel), or iPINP <50 µg/L (IDS iSYS) or TRACP5b <4.0 U/L.
- A high bone turnover state may be suspected if BALP is >35 µg/L (IDS iSYS) or 40 U/L (Quidel), iPINP >120 µg/L (IDS iSYS) or TRACP5b >5.0 U/L (IDS iSYS).
- Consider the possibility of a mineralisation defect if BALP levels are very high, particularly in the setting of insufficient calcium intake, vitamin D deficiency, or hypophosphataemia.

### Rationale

BTM correlate with other measures of skeletal remodelling, such as histomorphometry of bone biopsies [38] and radiotracer kinetics [39]. Correlations between BTM and bone histomorphometric indices of skeletal remodelling are modest in postmenopausal women [38] and BTM are not used for diagnostic purposes in postmenopausal osteoporosis. However, in CKD, there is a greater range of disturbances of bone turnover and therefore variability in BTM levels are more likely to be informative of the underlying bone phenotype.

### Diagnostic accuracy compared with a bone biopsy

**Figure 2:**
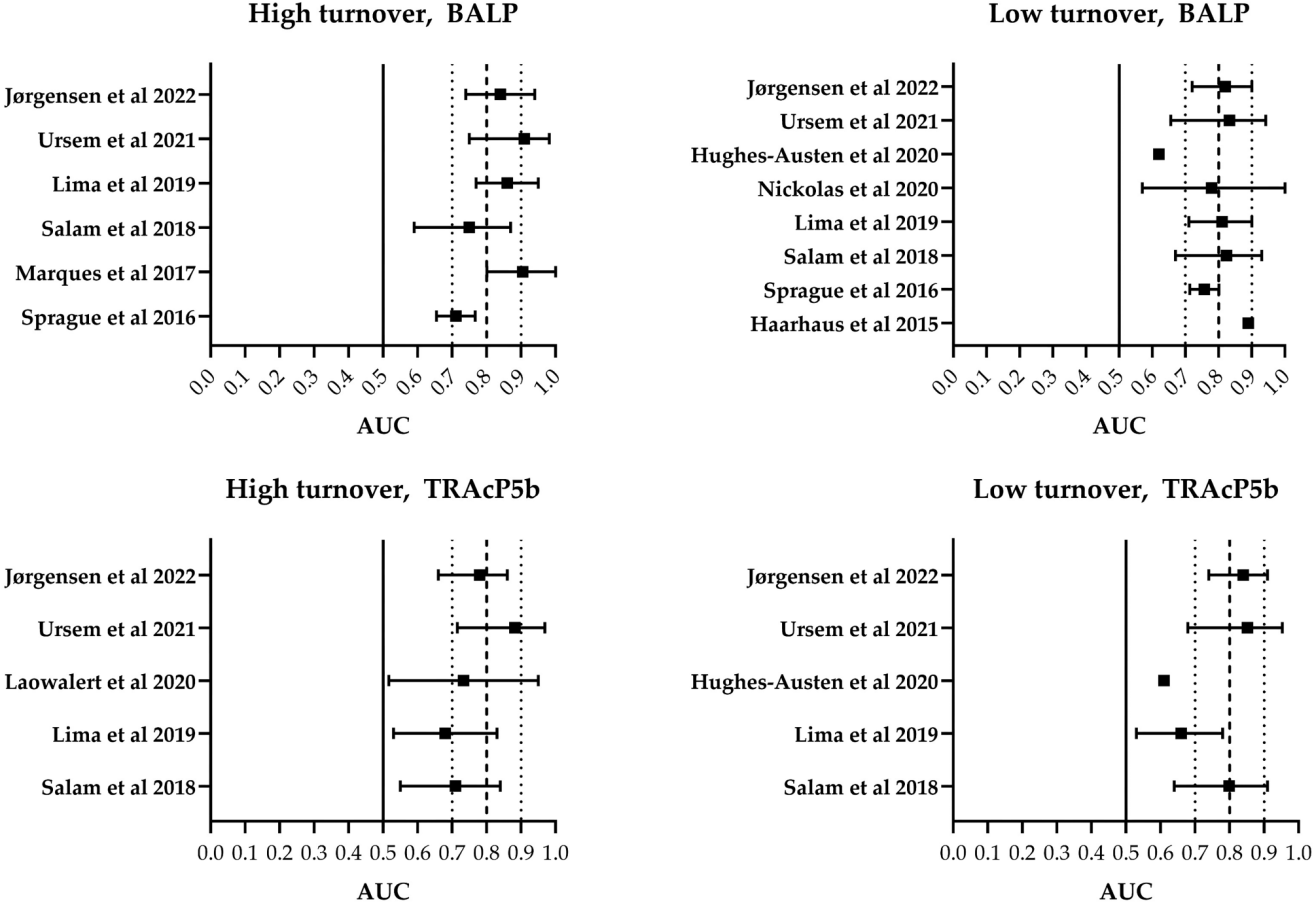
Diagnostic accuracy of BALP and TRACP5b in CKD with AUCs for diagnoses of high or low bone turnover by bone histomorphometry. Created with Graphpad Prism.

Studies comparing BTM and bone biopsy findings reveal moderately strong correlations between BTM and key histomorphometric variables such as the bone formation rate [40, 41]. Furthermore, non-kidney cleared BTM show good diagnostic performance for a diagnosis of high and low bone turnover as determined by bone biopsy, with AUC values in the range of 0.70–0.90 (Fig. 2) [40–47]. Although limited by the lack of consensus on histomorphometric reference values and the use of different biochemical assays, cutoffs for high and low turnover are fairly consistent between studies (Table 2). Based on current data it seems reasonable to suspect low bone turnover with BALP levels below 20–25 µg/L when measured by IDS-iSYS or 30–35 U/L when measured by the Quidel assay. Conversely, high bone turnover may be suspected with BALP levels above 30–35 µg/L, as measured by IDS-iSYS, or 35–40 U/L as measured by the Quidel assay—or with TRACP5b levels above 4.5–5.0 U/L. So far, only two studies reported on the diagnostic accuracy of intact PINP, both using the IDS-iSYS assay, indicating cutoffs of below 50–60 µg/L for low bone turnover and above 110–120 µg/L for high bone turnover. While sensitivity and specificity for the proposed cutoff levels for single BTM or combinations were generally above 70% in studies comparing BTM vs histomorphometry, positive predictive values for low bone turnover were lower than for high turnover, possibly reflecting a proportion of patients with non-low bone turnover and low levels of BTM.

**Table 2:** Proposed diagnostic cutoffs of BTM for high and low bone turnover in CKD.

	Study	Population	Assay	BTM reference range	Cutoff low	Cutoff high
BALP	de Oliveira 2015 [48]	CKDG5D (PD), *N* = 41	Alkphase™Metra Biosystem	Men 15.0–41.3 U/L; premenopausal 11.6–29.6 U/L; postmenopausal 14.2–42.7 U/L		>57 U/L
	Marques 2017 [49]	CKDG5D (HD), *N* = 31	Alkphase™Metra Biosystem			>66 U/L
	Sprague 2016 [43]	CKDG5D (HD), *N* = 450	Alkphase™Quidel		<33 U/L	>42 U/L
	Lima 2019 [44]	CKDG1–5D, *N* = 104	Alkphase™Quidel		<27 U/L	>35 U/L
	Salam 2018 [40]	CKDG4–5D, *N* = 43	Ostase^®^ BAP, IDS-iSYS	Men 7.9–25.5 µg/L; premenopausal 6.1–22.2 µg/L; postmenopauasl 7.1–23.9 µg/L	<21 µg/L	>31 µg/L
	Jørgensen 2022 [41]	CKDG3–5D, *N* = 199	Ostase^®^ BAP, IDS-iSYS		<24 µg/L	>34 µg/L
TRACP5b	Lima 2019 [44]	CKDG2–5D, *N* = 104	MicroVue™, Quidel	Men 4.0 ± 1.4 U/L; premenopausal 2.9 ± 1.4 U/L; postmenopausal 4.3 ± 1.5 U/L	<4.3 U/L	>4.3 U/L
	Salam 2018 [40]	CKDG4–5D, *N* = 43	BoneTRAP^®^, IDS-iSYS	Men 1.4–6.1 U/L; premenopausal 1.2–4.8 U/L; postmenopausal 1.1–6.9 U/L	<4.6 U/L	>4.6 U/L
	Jørgensen 2022 [41]	CKDG3–5D, *N* = 199	BoneTRAP^®^, IDS-iSYS		<3.4 U/L	>5.1 U/L
iPINP	Salam 2018 [40]	CKDG4–5D, *N* = 43	IDS-iSYS	Men 12.8–71.9 ng/mL; premenopausal 13.7–71.1 ng/mL; postmenopausal: <82.6 ng/mL	<57 µg/L	>107 µg/L
	Jørgensen 2022 [41]	CKDG3–5D, *N* = 199	IDS-iSYS		<50 µg/L	>121 µg/L
tPINP	Sprague 2016 [43]	CKDG5D (HD), *N* = 450	Elecsys^®^, Roche	Men: 13.9–85.5 ng/mL; premenopausal: 15.1–58.6 ng/mL; postmenopausal: 20.3–76.3 ng/mL	<499 µg/L	>621 µg/L
	Salam 2018 [40]	CKDG4–5D, *N* = 43	Elecsys^®^, Roche		<124 µg/L	>142 µg/L

HD, haemodialysis; PD, peritoneal dialysis.

BALP and TRACP5b are the BTM most extensively studied in the context of renal osteodystrophy [4]. BALP is also a marker of mineralisation, which can sometimes compromise its use as an indicator of bone formation. BALP levels will (transiently) rise in situations of rapid mineralisation, such as in the hungry bone state induced by parathyroidectomy [50, 51] or other potent PTH lowering therapies [52]. Although BTM are not diagnostic of bone mineralisation defects, osteomalacia should be considered with very high levels of BALP. The evidence related to bone mineralisation defects is sparse, particularly for contemporary cohorts, but one study reported BALP (measured with the MicroVue EIA assay; Quidel) levels of median 88.5 U/L (interquartile range 52.5–92.8) in patients with abnormal mineralisation versus 31.7 U/L (interquartile range 25.2–38.9) in those with normal bone mineralisation. [53]. A mineralisation defect should be suspected if high BALP levels coincide with clinical symptoms, e.g. bone and joint pain, and/or predisposing factors, e.g. vitamin D deficiency, hypophosphatemia or hypocalcaemia.

**Figure 3:**
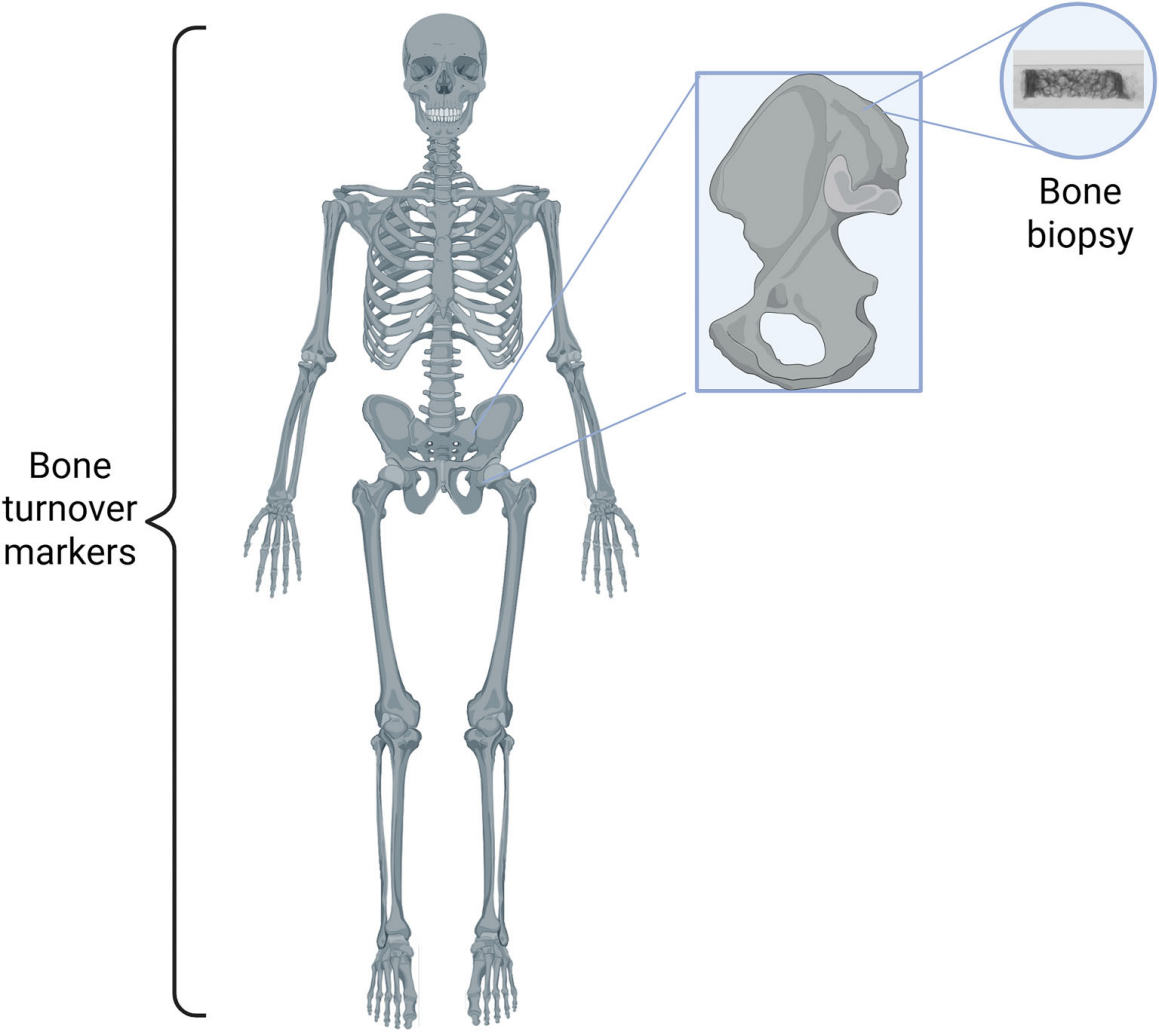
Sample size of a bone biopsy versus bone turnover markers. Bone histomorphometry informs on skeletal remodelling, mineralization and microarchitecture in a small sample from the iliac crest, while BTM reflect the total skeleton, including both cortical and trabecular bone. Created with BioRender; bone sample image courtesy of Dr Syazrah Salam, Sheffield, UK.

The anterior iliac crest bone biopsy remains the gold standard to evaluate the metabolic bone component of CKD-associated osteoporosis, and most studies investigated the diagnostic accuracy of BTM against this standard. However, the comparison of these two methods deserves some deliberations. A bone biopsy delivers highly detailed information on skeletal remodelling, mineralisation, and microarchitecture, but is limited by a small sample size, and thus is susceptible to sampling bias [54]. Furthermore, the histomorphometric analysis is traditionally performed on trabecular bone, although cortical bone deficits are highly common in CKD-associated osteoporosis [55, 56]. In contrast, BTM are measured in the systemic circulation and must therefore represent the total skeleton, including both trabecular and cortical bone compartments (Fig. 3). There is also a time-lag present when comparing results from a bone biopsy and BTM; findings on bone histomorphometry represent what has occured in the bone over the last 3–6 months, while BTM are highly dynamic, representing the current status quo [57]. Thus, one could conclude that these two methods deliver different information regarding the state of skeletal remodelling. Finally, there is currently no consensus on the appropriate normal reference range for bone histomorphometric variables, resulting in different diagnostic cutoffs for high and low bone turnover. This contributes to heterogeneity between studies and is a clear limitation to the use of bone histomorphometry as the gold standard. An alternative approach could be to apply the normal reference for the general population, for example the median level of premenopausal women or young men, and define high and low bone turnover as deviations of BTM from these ranges [58]. Target range could also be defined based on the strength of association with prospective outcomes; however, evidence to support these approaches is not available for CKD-associated osteoporosis.

### BTM and risk prediction

*Key evidence points*

- Higher levels of BTM indicate increased risk of bone mineral density (BMD) loss, at least at the population level.
- Higher levels of BTM indicate increased risk of incident fractures, at least at the population level.

*Clinical practice points*

- High and increasing levels of BTM indicate increased risk of BMD loss and should be addressed.
- High levels of BTM indicate increased fracture risk, but current evidence is insufficient to define thresholds for individual fracture risk assessment in routine clinical practice.

### Rationale

BTM are increasingly used for monitoring of treatment response, including assessment of adherence to medication. However, even though higher levels of BTM are associated with incident fracture risk, current evidence is limited regarding fracture prediction on an individual level in postmenopausal osteoporosis [59]. The value of BTM as risk predictors may be different in the setting of CKD, as disturbances of skeletal remodelling are much more prominent in CKD-associated versus postmenopausal osteoporosis. In CKD, higher levels of BTM indicate a high bone turnover state, with excess bone resorption [60]. Concurrently, higher levels of BTM associate with lower BMD by dual-energy X-ray absorptiometry (DXA) and greater prevalence of fracture both in early (CKDG2–5) [61, 62] and late (CKDG5D) [63–66] CKD. Higher baseline and time-averaged BTM levels associate with higher rates of bone loss over time, particularly at the cortical skeleton [56, 64].

**Figure 4:**
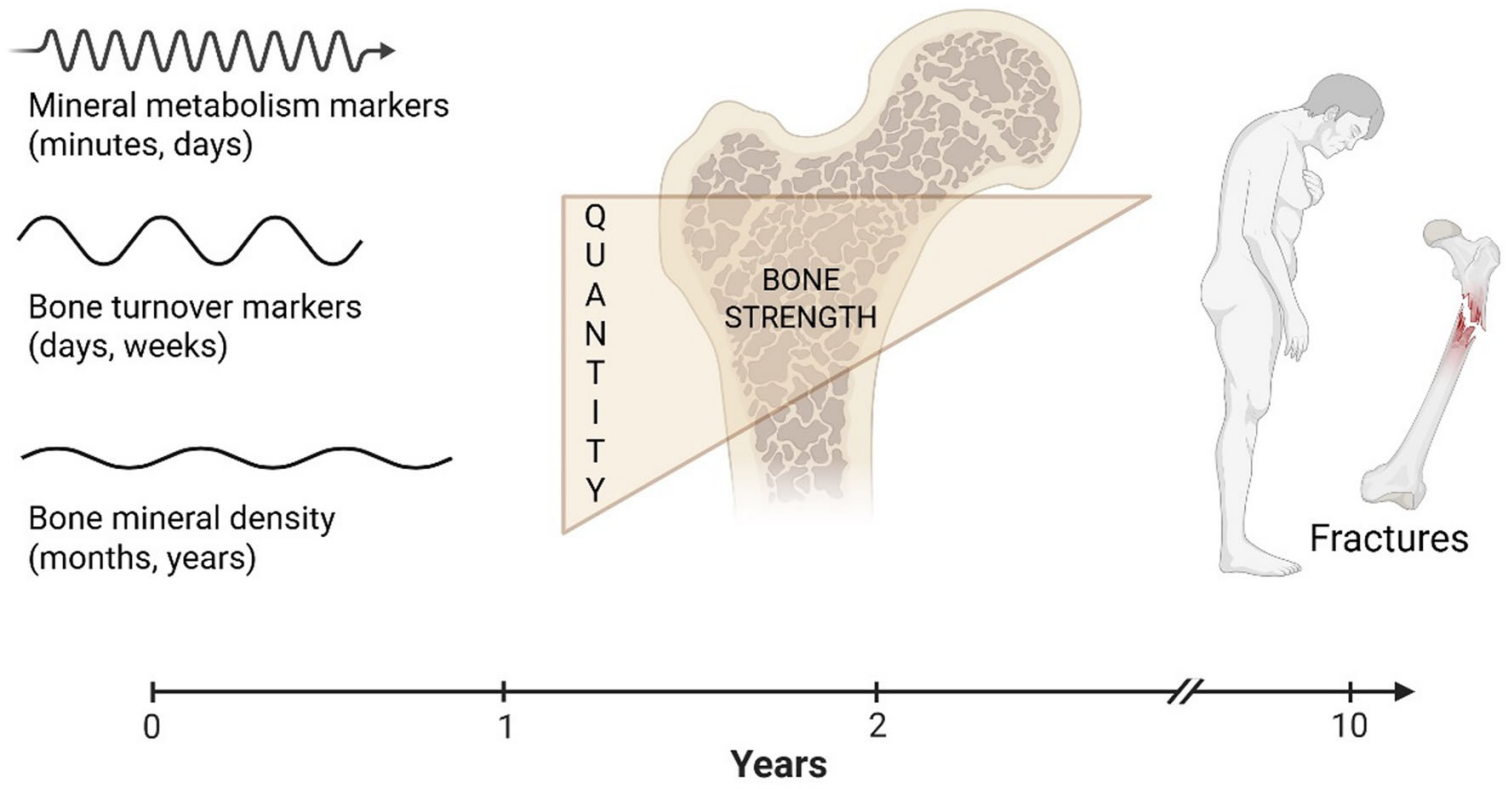
Mineral metabolism disturbances affect skeletal remodelling which, in turn, may cause bone loss due to high bone resorption. Thus, markers of mineral metabolism are useful to consider the contributors to deviations in bone turnover in CKD-associated osteoporosis. BTM reflect the current status of skeletal remodelling and can be measured serially to monitor changes. All biomarkers are highly dynamic, changing rapidly in response to changing conditions (including therapy); this may hamper their usefulnes in predicting clinically relevant outcomes such as bone loss (months to years of exposure) and ultimately fractures. Created with BioRender.

The ability of BTM to predict fractures in CKD has only been assessed by a handful of studies (Table 3). Higher levels of BALP [67], or total ALP as a surrogate or proxy for BALP [68–70], associate with incident fracture risk, at least in patients with CKDG5D receiving dialysis. Higher levels of ALP also associate with prevalent vertebral fractures [66], but studies investigating the ability of biomarkers to predict incident vertebral fractures are lacking. These fractures are often asymptomatic and are likely to be underdiagnosed in CKD [71]. BTM measured at time of kidney transplantation did not predict fractures in the long-term post-transplant [72], presumably due to the nature of BTM, reflecting the ‘current’ skeletal remodelling status of the total skeleton, while kidney transplantation can induce prominent, divergent and long-lasting changes in the skeleton [73]. Sustained high levels of bone resorption markers are likely to indicate ongoing bone resorption and bone loss—which, in the long-term, may negatively affect bone quantity and quality, resulting in bone fragility (Fig. 4). However, neither single time-point measurements of BTM nor trends over time have been sufficiently studied to establish them as markers of long-term fracture risk in the individual patient. The post-transplant period is characterised by marked changes in mineral metabolism as well as the influence of immunosuppressants, and under such dynamic conditions, serial measurements of BTM are likely to be more informative [74]. In keeping with this, Iimori and colleagues found that BALP levels at the nearest timepoint before a fracture event was the best predictor of incident fractures in patients receiving haemodialysis [67]. Interestingly, their BALP cutoff for increased fracture risk was fairly low (>20 µg/L; Access Ostase^®^ assay, Beckman Coultier, Inc.), which is in line with the somewhat lower results reported using this method, compared with the IDS ISYS and Quidel Micro Vue in a previous comparative study [35].

**Table 3:** Cohort studies investigating the value of BTM for fracture risk prediction in CKD.

Study	Population	Design	Biomarker	Outcome	Findings
Maruyama 2014 [68] (JSDT)^a^	CKD5D, *N* = 185 277	Retrospective, longitudinal	ALP	Mortality, CVD mortality, hip fracture	Higher ALP associated with mortality, CVD, and fracture
Maruyama 2025 [70] (JSDT)^a^	CKD5D, *N* = 241 670	Retrospective, longitudinal	ALP	Mortality, CVD mortality, hip fracture	Higher ALP associated with mortality and fracture, marginally associated with CVD mortality
Yamamoto 2024 [69] (DOPPS)	CKD5D, *N* = 28 888	Retrospective, longitudinal	ALP	Mortality, CVD mortality, Fracture	Higher ALP associated with mortality, CVD, and fracture
Chang 2021 [75]	CKD5D, *N* = 352	Prospective, longitudinal	ALP	Fracture	Higher ALP associated with incident fracture
Iimori 2012 [67]	CKD5D, *N* = 458	Prospective, 5 years	BALP	BMD, fracture	Higher and increasing levels of BALP associated with bone loss and fracture
Evenepoel 2019 [72]	CKDG5D, *N* = 518	Prospective, 10 years	BALP, iPINP, TRACP5b	Fracture	No association between BTM at time of kidney TX and long-term fracture risk
Matias 2020 [76]	CKDG5D, *N* = 341	Retrospective, longitudinal	BALP	Fracture	Higher BALP associated with fracture
Nakagawa 2022 [65]	CKDG5D, *N* = 654	Prospective, 3 years	BALP, TRACP5b	BMD, fracture	Higher BALP and TRACP5b associated with fracture

^a^Updated dataset from the same registry. CVD, cardiovascular disease; DOPPS, Dialysis Outcomes and Practice Patterns Study; JSDT, Japanese Society for Dialysis Therapy; TX, transplant.

In general, studies investigating the predictive ability of BTM in the setting of CKD were mainly retrospective and often based on large dialysis registries [68–70]. These studies typically utilised ALP as a surrogate for BALP, with inherent limitations. In addition to potential interference from liver ALP [20], inflammation may also upregulate alkaline phosphatases of various origins [27, 77]. A recent study indicated that the ‘non-skeletal’ contribution to total ALP may be a driver of poor outcomes both in a non-CKD population and in early stages of CKD [28]. So far, there are no studies on the predictive ability on BTM on bone outcomes in moderate (pre-dialysis) CKD. Thus, while BTM, particularly when measured serially, show promise as risk markers in CKD-associated osteoporosis there is currently insufficient evidence to recommend the implementation of BTM for individual risk assessment in routine clinical practice.

### BTM as treatment targets

*Key evidence points*

- Circulating levels of BTM at initiation of bone-targeting treatments predict clinical efficacy as well as the risk of hypocalcaemia with antiresorptive treatment.
- In bone-targeting interventions, change in BTM after treatment initiation predict clinical efficacy.

*Clinical practice points*

- BTM should be determined before treatment initiation to guide choice of treatment in CKD-associated osteoporosis.
- Assessment of BTM should be integrated into monitoring the efficacy of bone targeting therapies.
- A treatment response in bone is indicated by a change in BTM greater than the LSC.
- A failure of BTM to respond to therapy should prompt considerations of treatment failure (including inadequate dosing regimen or non-compliance).

### Rationale

CKD-associated osteoporosis can present with a range of disturbances in bone turnover and mineralisation, which needs to be taken into account when deciding on a therapeutic strategy. Non-skeletal causes of severe bone turnover disturbances, e.g. severe high-turnover hyperparathyroidism, or greatly decreased bone turnover due to oversuppression of PTH, malnutrition and/or inflammation, should be addressed before initiating anti-osteoporosis treatment. Treating severe hyperparathyroidism with calcimimetics [78] or parathyroidectomy [79] can improve BMD, while the effect on fracture risk is less well documented [80–83]. Improving nutritional status, especially when combined with exercise [84], also has the potential to improve bone strength even in late-stage CKD [85].

The baseline level of BTM has been shown to predict therapeutic response. BTM are therefore prime candidates as treatment targets in CKD-associated osteoporosis. Higher baseline BTM [86–92], as well as greater decrease in BTM during treatment [86], are associated with greater increases in BMD during anti-resorptive therapy in observational studies (Table 4). At the same time, however, high BTM and PTH at initiation of anti-resorptive therapy indicate a high risk of hypocalcaemia [88–93]. Both may be related to a temporary imbalance in bone formation and resorption in favour of the former, translating to a positive bone calcium balance. Active remineralisation, often referred to as the ‘hungry bone response’, will be most pronounced in patients with high-turnover hyperparathyroid bone disease. Hypocalcaemia will manifest in those individuals in whom the exogenous calcium supply (through oral intake or dialysate) is insufficient to compensate for the positive bone calcium balance [94]. This relationship between BTM and treatment and adverse effects are best documented for denosumab, while bisphosphonates, which are off-label in late-stage CKD, are likely to be associated with less pronounced BMD increase [95] as well as a lower risk of hypocalcaemia [96]. The responses of BTM and circulating calcium levels are not limited to drugs that directly target bone—any therapy that affects bone metabolism will be reflected in changes in the BTM and may influence calcium levels. Targeting severe hyperparathyroidism with potent calcimimetics leads to a marked decreases in BTM and may induce hypocalcaemia [52], and high levels of BALP, or ALP as a surrogate of BALP, predict a hungry bone response after parathyroidectomy [97–100]. Both strategies have the potential to improve BMD. By inhibiting the ability of bone to increase resorption in response to PTH, anti-resorptive treatment often cause an increase in circulating PTH. Thus, based on these observations (despite lack of direct evidence) it is advisable to target severe hyperparathyroidism as an underlying cause of increased BTM to mitigate the risk of hypocalcaemia and rebound hyperparathyroidism and to consider re-evaluating BMD before starting anti-resorptive therapies. In patients with low to moderately increased bone turnover, the hypocalcaemia risk with anti-resorptive treatment still exists, but can usually be adequately addressed by preemptive treatment with calcium and vitamin D [94], and initiation of antiresorptive therapy should not be delayed.

**Table 4:** BTM in monitoring therapeutic response and adverse treatment effects in CKD.

Study	Population	Intervention	Biomarker	Conclusion
Fujii 2007 [86]	CKDG1–3, *N* = 114	R IS, 12 months	BALP, NTX, OC	BTM changes predict treatment effect
Miyaoka 2019 [88]	CKDG1–3b, *N* = 77	DMAB, 6 months	BALP, TRACP5b	BTM at baseline predict treatment adverse effects
Kim 2024 [101]	CKDG3b–4, *N* = 20	DMAB, 12 months	BALP, β-CTX-I	No value of BTM in predicting treatment effect
Horikawa 2022 [92]	CKDG3–5, *N* = 52	DMAB, 12 months	tPINP, TRACP5b	BTM at baseline predict treatment adverse effects
Kunizawa 2020 [89]	CKDG1–5D, *N* = 324	DMAB, 12 months	BALP, tPINP, TRACP5b	BTM at baseline predict treatment and adverse effects
Hiramatsu 2021 [90]	CKDG5D, *N* = 47	DMAB, 24 months	BALP, TRACP5b	BTM at baseline predict treatment and adverse effects
Hori 2022 [91]	CKDG5D, *N* = 28	DMAB, 6 months	BALP, TRACP5b	BTM at baseline predict treatment and adverse effects
Sumida 2016 [102]	CKDG5D, *N* = 11	TERI, 11 months	BALP, PINP, TRACP5b	BTM at baseline predict treatment effect
Saito 2023 [103]	CKDG5D, *N* = 13	ROMO, 12 months + DMAB 12 months	TRACP5b, PINP	BTM at baseline predict treatment effect

DMAB, denosumab; NTX, N-terminal telopeptide; OC, osteocalcin; ROMO, romozosumab; TERI, teriparatide.

For patients with low bone turnover, anti-resorptive therapies appear less efficacious [90, 91]. There is also a theoretical concern in suppressing already low turnover further, as very low bone turnover has been implicated in bone fragility, e.g. atypical femoral fractures [104]. However, it is important to highlight that we do not have strong evidence of harm from anti-resorptive drugs in low bone turnover states. In non-high turnover states, the bone calcium balance may be a better predictor of the therapeutic response than bone turnover *per se*. In patients with low BTM, indicative of low bone turnover, anabolic treatment has been shown to improve BMD in CKD [102,103,105–107], although studies are small and evidence for fracture risk reduction is missing. Of interest, higher BTM indicate greater likelihood to respond to bone anabolic therapy as well [102].

The non-invasive diagnosis of impaired mineralisation remains a clinical challenge, although very high levels of BALP can indicate osteomalacia. For a definitive diagnosis of a bone mineralisation defect, a bone biopsy is still necessary. Despite low prevalences of osteomalacia in contemporary cohorts of kidney failure [53, 73], it is an important differential diagnosis when considering treatment of CKD-associated osteoporosis, as anti-resorptive therapies should not be applied. A pragmatic approach to this issue would be to optimise the reversible causes of bone mineralisation defects by avoiding metabolic acidosis and deficiencies in calcium, phosphate and vitamin D prior to initiating bone-targeting therapies and monitor the effect on BALP or total ALP.

BTM show a rapid and pronounced decline after initiation of anti-resorptive therapy in CKD [89, 90, 93], which is no different from that of non-CKD patients [89]. Following the potent anti-resorptive denosumab, bone resorption markers decrease within weeks, while bone formation markers follow within the next months [89], due to the fact that bone formation and resorption are coupled. A rise of BTMs following the initial suppression after a denosumab injection can indicate treatment escape, which may occur within the recommended dosing interval. Failure to administer denosumab at the time of increasing BTMs can decrease treatment efficacy and increase fracture risk [108]. While weekly teriparatide injections in CKD induced an increase in bone formation markers and a concomitant decrease in bone resorption markers [105, 102], a biochemical pattern termed the anabolic window, it is unclear whether other treatment intervals may induce a different BTM response. Thus, BTM can be used to assess the response to treatment early in the treatment course. A failure of BTM to respond should prompt investigation into the cause—including the possibility of lack of adherence to the therapy in question—and could warrant a bone biopsy.

### Paediatric considerations

*Key evidence points*

- Due to the inherent physiological differences in skeletal remodelling and mineralisation, studies in adults cannot be extrapolated to children.
- There are only limited data for paediatric reference values for most BTM.
- The utility of BTM for the diagnosis and prognosis of bone health remains to be fully investigated, both in healthy children and in those with CKD.

*Clinical practice points*

- Consider age-related, and if appropriate, sex-specific normal ranges for all biomarkers.

### Rationale

Due to the inherent physiological differences in skeletal remodelling and mineralisation, studies related to CKD-MBD or CKD-associated osteoporosis in older adults cannot be directly extrapolated to children, teenagers, and young adults who have not yet reached peak bone mass. In a similar fashion, even though evidence is scarce, BTM must be approached separately, and comprehensively understood to be applied to clinical practice in paediatrics.

Bone formation markers proposed for routine use in children include BALP and iPINP. BALP rises in childhood, particularly during periods of rapid growth during infancy and puberty, is higher in boys, and has been associated with growth velocity [109–111]. In routine clinical practice, ALP is used as a surrogate marker of BALP, unless there is severe liver disease. For both bone formation markers, however, paediatric data are limited. Reference ranges are available for healthy children [112–114], but most of these studies lack relevant clinical data including pubertal status, and have measured a limited range of BTM only. More studies are needed to validate iPINP as a reliable marker of bone formation in paediatric CKD [111, 115, 116]. The bone resorption marker TRACP5b is not associated with CKD grade but did closely associate with PTH levels, and was lower in children treated with recombinant human growth hormone [109], and may thus be a promising marker for skeletal health in paediatric CKD.

BALP, iPINP, and TRACP5b may offer non-invasive insight into bone health in paediatric CKD. All these markers can aid diagnosis and monitoring when used in context, but paediatric-specific reference data are limited. Thus, most BTM require more validation in healthy children and subsequently in children with CKD. Clinicians should follow recent CKD-MBD guidelines from KDIGO [3] and the ESPN [117, 118] which stress monitoring trends in values, focusing on PTH and calcium-phosphate control, and judicious use of BTM rather than relying on any single value. Further studies are needed to understand the role of BTM in predicting fractures in paediatric CKD.

### Integrating BTM into clinical practice

*Clinical practice points*

- Routine monitoring of BTM should be considered from CKDG3 onwards with reasonable intervals of 12 months in CKDG3, 3–6 months in CKDG4 and 3 months in CKDG5–5D.
- Beware of expected increases of BTM up to 6–12 months after a fracture.
- BTM may replace a bone biopsy for the evaluation of bone turnover in many clinical settings.
- BTM can be supportive but cannot replace DXA for fracture risk assessment.

### Rationale

As detailed in previous sections, BTM may be useful for risk assessment, establishing bone phenotype, optimising mineral metabolism, determining the therapeutic strategy, and monitoring of treatment effects in CKD-associated osteoporosis. However, the question arises on how clinicians can integrate BTM into the available spectrum of diagnostic tools and treatment strategies, comprising other biochemical markers, bone biopsy with histomorphometry, and DXA. The value of BTM in relation to specific diagnostic tools is discussed below.

### Frequency of BTM monitoring

Routine monitoring of BTM allows for assessment of trends over time, which is more informative than single time-point measurements considering the dynamic nature of the underlying bone metabolism disturbances in CKD. Previous KDIGO recommendations on frequency of BTM monitoring in the context of CKD were restricted to total ALP with low frequency and BALP as an alternative to PTH, while PTH levels above the target were suggested to trigger a higher frequency of ALP monitoring. PTH monitoring was recommended with increasing frequency as CKD progresses despite analytical issues, high variability, and an impractically wide target range defined in the guideline. On the contrary, ALP and BALP lacked target ranges and thus, are seldom used as routine markers in clinical practice. The definition of diagnostic cutoffs for BALP, iPINP, and TRACP5b in this consensus strengthens their clinical applicability. Importantly, knowledge of bone turnover status and its dynamic change can have a direct impact on treatment choices. Therefore, we recommend monitoring BTM either as adjunct or alternative to PTH, which can be monitored at a lower frequency in routine clinical practice or merely be used for the diagnostic workup and management of high bone turnover in case of increased BTM. Pragmatically, the monitoring intervals previously suggested for PTH could be applied, with measurements of BTM every 3–6 months for patients with CKDG4–5D [119]. If difficult to obtain due to availability or cost issues, ALP may be an acceptable substitute, allowing for less frequent measuring of the specific BTM. The finding of increased BTM could indicate a need of PTH suppression. On the other hand, low BTM could initiate evaluation of nutritional status, inflammation, and other modifiable causes of low bone turnover. Patients being exposed to potent PTH suppressive or antiresorptive therapy may benefit from more frequent monitoring, e.g. to guide calcium supplementation. The advantage of BTM over PTH for the evaluation of bone turnover is highly relevant in antiresorptive treatment, where PTH often increases and dissociates from bone turnover, while BTM reflect the actual treatment effect on bone. Basing treatment choices on PTH may be inadequate in this situation.

A particular concern for therapeutic decisions in CKD-associated osteoporosis is the increase in BTM immediately following a fracture, which is evident within weeks and can last for up 6–12 months [57]. Having an established trajectory of BTM leading up to the fracture event could counteract this limitation.

### BTM and bone biopsy

Historically, a bone biopsy with histomorphometric analysis has been recommended with varying indications, often emphasising the clinical impact of knowledge of bone turnover. As bone histomorphometry expertise is rapidly waning, and given its invasive nature, bone biopsies are poorly suited for routine evaluation in everyday clinical practice, especially in the setting of longitudinal follow-up. The diagnostic cutoffs recommended in the current consensus render BTM suitable for a diagnosis of bone status in most clinical settings. Thus, we recommend restricting bone biopsies to situations with unexplainable discrepancies between BTM or when a mineralisation defect is suspected but cannot be managed based on clinical indicators and BTM alone.

### BTM and DXA

In postmenopausal osteoporosis, DXA is superior to BTM in individual fracture risk prediction. While some studies suggest that, due to the more severe bone turnover abnormalities, BTM may have a modifying effect on the fracture risk prediction by DXA, or even outperform DXA, in CKD [67], there is still a scarcity of data on individual fracture risk prediction by BTM in CKD. Thus, current findings from non-CKD patients are extrapolated to CKD patients, awaiting further evidence. DXA BMD should thus be the primary basis for treatment decisions regarding fracture risk reduction [120]. As patients with CKDG4–5D can be considered at especially high fracture risk, screening of postmenopausal women and individuals aged >50 years has been suggested while secondary fracture risk prevention after a major osteoporotic fracture can be started without consideration of a DXA BMD threshold [120].

### Results of the Delphi survey

The Delphi survey, detailing the clinical practice points developed during the consensus process, was returned by 29 clinical experts in CKD-associated osteoporosis, not involved in writing of the current manuscript. Overall, there was a high level of agreement for all clinical practice points with >70% of experts agreeing or strongly agreeing with the statements. Agreement was generally 80%–90%. There was a >5% disagreement with four statements, most notably, 21% of responders disagreed that bone biomarker could replace a bone biopsy. These statements were revised and re-approved by all co-authors for the final manuscript. Details of the statements in the Delphi survey and responses can be found in Supplementary data, Fig. S1.

### Research recommendations

#### Bone balance index

In physiological bone remodelling, bone resorption and formation are tightly coupled. Pathological conditions and therapeutic interventions however, may cause both processes to be uncoupled. It is suggested that combining the measurements of bone resorption/osteoclast and formation/osteoblast markers may provide information about the bone balance [121]. Although conceptually attractive, the diagnostic yield and benefit of the bone balance index, i.e. a metric integrating bone formation and resorption markers, remains to be determined. A negative bone balance index could identify patients at risk for bone loss and fracture, but further validation studies are required, particularly in the setting of CKD.

#### BTM and clinical risk prediction tools

Clinical risk prediction tools have been validated for patients with CKD, where they perform as well as in the general population. However, their specific role in the management of CKD-associated osteoporosis has yet to be established. In addition, the added value of combinations of clinical risk prediction tools with DXA and/or BTM in CKD requires further investigation.

#### BTM vs other non-invasive measures of bone turnover

Other methods of evaluating skeletal bone remodelling include radiotracer and calcium isotope techniques. ^18^F-NaF-PET combines a bone-seeking tracer with high-resolution imaging and delivers estimates of global and regional bone turnover, with a good agreement when compared with bone histomorphometry [122, 123]. Very few studies investigated the relationship between ^18^F-NaF-PET and BTM in CKD, and so far, none has reported on diagnostic accuracy [124–126]. Naturally occurring stable Ca isotopes have been suggested as non-invasive markers of bone balance. The ratio between the lighter isotope (^42^Ca) which preferentially seeks bone, and the heavier isotope (^44^Ca) which remains in the blood, increases with bone formation and decreases with bone resorption, and can be considered a measure of skeletal remodelling [127–129]. The Ca isotope ratio (δ^44/42^Ca) correlates with BTM in children and adults with CKD [130]. Further research is needed to understand the information to be gained from radiotracer and calcium isotope techniques in comparison to bone histomorphometry and BTM, both in the non-CKD population and in the setting CKD-associated osteoporosis.

#### Treatment target definition

The association of BTM with clinical fracture risk and risk of other adverse outcomes, their robustness as biomarkers and general availability renders them ideal as clinical treatment targets. Further research should therefore focus on identifying individual risk prediction thresholds and adequate target levels for risk reduction. More specifically, determination of the reference BTM, TRACP5b and BALP, should be included in all future clinical trials targeting bone turnover [4]. In addition, specific treatment targets should be evaluated in clinical trials.

#### CONCLUDING REMARKS

The high fracture risk associated with CKD is insufficiently addressed by established risk markers and current treatment strategies for bone and mineral disorders in CKD. Introducing the term ‘CKD-associated osteoporosis’ has shifted the focus from a PTH-centric approach to the skeleton as target organ, enabling novel approaches to the management of skeletal complications in CKD. This shift requires the introduction of easily applicable bone status indicators that can render sufficient diagnostic and predictive accuracy for clinical decision making. The non-kidney cleared BTM, BALP, TRACP5b, and iPINP, are reliable tools for bone evaluation in CKD-associated osteoporosis and methods are available for use in clinical laboratories, however accessibility in routine care is variable and needs to be improved. The current consensus establishes their clinical usefulness in diagnosis of ROD, fracture risk prediction, choice of therapeutic strategy and treatment evaluation. Future research should focus on the integration of BTM with other diagnostic tools, the establishment of treatment targets, and development of pharmacological and non-pharmacological strategies to reach these targets.

## Supplementary Material

gfag033_Supplemental_File

## Data Availability

No new data were generated or analysed in support of this research.
