# Supplementary material for: Bone turnover markers in the management of CKD-associated osteoporosis—a European consensus
Source: Nephrol Dial Transplant. 2026 Feb 17;41(7):1357–75. doi: 10.1093/ndt/gfag033 (PMC13314378; doi:10.1093/ndt/gfag033)

**Supplementary Material**

Table S1 Survey of the clinical use of bone turnover markers in CKD-associated oste-oporosis

Table S2 Results of survey on the availability of bone turnover markers

Table S3 Literature search strategies of the consensus

Figure S1 Results from the Delphi survey on suggested clinical practice points of the consensus

**Table** S1. Survey of the clinical use of bone turnover markers in CKD-associated oste-oporosis

What is your role? (multiple answers possible)

If you chose "Other clinical specialist", please specify:

What is your age?

What setting do you work in?

Do you treat osteoporosis in your patients?

*Do you have access to the following imaging techniques:*

- Dual-energy x-ray absorptiometry (DXA)
- Peripheral quantitative computed tomography (pQCT)
- High-resolution peripheral quantitative computed tomography (HR-pQCT)
- 18F sodium fluoride positron emission tomography (18F NaF-PET)
- Please specify other imaging available and whether accessible in clinic or re-search only

*Do you have access to:*

- Bone histomorphometry
- Other bone histology

*Which biomarkers do you have access to:*

- Total alkaline phosphatase (tALP)
- Bone-specific alkaline phosphatase (BALP)
- Total (monomeric) pro-collagen type I N-terminal propeptide (tPINP)
- Intact (trimeric) pro-collagen type I N-terminal propeptide (iPINP)
- C-terminal cross-links (CTX)
- Tartrate resistant acid phosphatase isoform 5b (TRAP5b)
- Please specify other bone turnover markers and whether accessible in clinic or research only

*Diagnostic approach*

Which routine biochemical markers do you use to identify low bone turnover?

- Multiple answers possible

Which routine biochemical markers do you use to identify high bone turnover?

- Multiple answers possible

Do you use imaging to identify high or low bone turnover?

- If you chose "Yes" please specify:

Do you use bone histomorphometry to identify high or low bone turnover?

Do you use histology without histomorphometry to identify high or low bone turnover?

Which routine biochemical markers do you use to identify bone mineralization de-fects?

- Multiple answers possible

Do you use imaging to identify bone mineralization defects?

- If you chose "Yes" please specify

Do you use bone histomorphometry to identify bone mineralization defects?

Do you use histology without histomorphometry to identify bone mineralization de-fects?

Do you use clinical symptoms and signs to identify bone mineralization defects?

- If you chose "Yes" please specify

*In what clinical settings do you use BTM:*

- For the evaluation of medical treatment of hyperparathyroidism:
- Before parathyroidectomy
- After parathyroidectomy
- Treatment of low bone turnover
- Treatment of bone mineralization defects
- Treatment of vascular calcification
- Evaluation of fracture risk in CKD
- Before initiation of antiresorptive treatment
- Evaluation of treatment effect of antiresorptive treatment
- After cessation of antiresorptive treatment
- Before initiation of anabolic treatment
- Evaluation of treatment effect of anabolic treatment
- After cessation of anabolic treatment
- Before kidney transplantation

*Do you use BTM as treatment targets:*

- In treatment of hyperparathyroidism?
- In treatment with antiresorptive agents?
- In treatment with anabolic agents?

| **Table** S2 Results of survey on the availability of bone turnover markers | | | | |
| --- | --- | --- | --- | --- |
|  | Own hospital | Other hospital | Research only | No access |
| *Imaging* |  |  |  |  |
| Dual-energy x-ray absorptiometry | 35 (76%) | 8 (17%) | 0 | 3 (7%) |
| Peripheral quantitative computed tomography (pQCT) | 10 (22%) | 3 (7%) | 8 (17%) | 25 (54%) |
| High-resolution pQCT | 11 (24%) | 6 (13%) | 10 (22%) | 19 (41%) |
| 18F-NaF-PET | 11 (24%) | 7 (15%) | 12 26%) | 16 (35%) |
| *Histology* |  |  |  |  |
| Bone histomorphometry | 12 (26%) | 15 (33%) | 7 (15%) | 12 (26%) |
| Bone histology | 10 (22%) | 9 (20%) | 9 (20%) | 18 (39%) |
| *Biomarkers* |  |  |  |  |
| Total alkaline phosphatase | 44 (96%) | 1 (2.2%) | 0 | 1 (2.2%) |
| Bone-specific alkaline phosphatase | 32 (70%) | 8 (17%) | 2 (4%) | 4 (9%) |
| Total pro-collagen type I N-terminal propeptide | 15 (33%) | 11 (24%) | 12 (26%) | 8 (17%) |
| Intact pro-collagen type I N-terminal propeptide | 10 (22%) | 15 (33%) | 11 (24%) | 10 (22%) |
| β-isomerized C-terminal telopeptide of type I collagen | 23 (50%) | 10 (22%) | 5 (11%) | 8 (17%) |
| Tartrate resistant acid phosphatase isoform 5b | 9 (20%) | 14 (30%) | 13 (28%) | 10 (22%) |
| Data shown as *n (%)* | | | | |

| Table S3 Literature search strategies of the consensus | |
| --- | --- |
| Methodological considerations | ((((tartrate OR tartrate-resistant) AND acid phosphatase 5b) OR (bone alkaline phosphatase OR bone-specific alkaline phosphatase) OR (intact AND (pinp OR p1np OR procollagen 1 propeptide OR procollagen i propeptide)) OR (bone turnover markers) OR (bone metabolic markers)) AND (chronic kidney disease OR ckd OR end-stage kidney disease OR end-stage renal disease OR esrd OR eskd OR chronic renal failure OR crf OR kidney failure OR hemodialysis OR dialysis)) AND humans.  In addition to the PubMed search, a manual review of the reference lists from the included studies and related reviews was conducted. Furthermore, two recent key reviews (Kidney Int (2025) 107, 405–423. Doi: 10.1016/j.kint.2024.11.013; Osteoporos Int (2025) 36(4):579-608. Doi: 10.1007/s00198-025-07422-3) were examined to identify additional relevant methodological information and to ensure the comprehensiveness of the literature search. |
| Diagnostic accuracy | (P1NP OR PINP OR ”bone ALP” OR bALP OR “bone alkaline phosphatase” OR BAP OR TRACP5b OR TRAP OR TRAP5b OR “bone turnover marker”) AND (“chronic kidney disease” OR dialysis OR hemodialysis OR “kidney transplantation” OR “renal insufficiency, chronic” OR “kidney failure”) NOT (review OR case report OR document OR book). Filter: Humans, English language  In addition to the PubMed search, a manual review of the reference lists from the included studies and related reviews was conducted. |
| Risk prediction | (bone turnover marker* OR alkaline phosphatase OR P1NP OR tartrate) AND (bone mineral density OR bmd OR fracture OR mortality OR survival) AND (kidney disease OR dialysis OR hemodialysis OR peritoneal dialysis). Filter: Humans, English language  Snowballing of the references in the retrieved papers was performed |
| Treatment target and evaluation | ((((tartrate OR tartrate-resistant) AND acid phosphatase 5b) OR (Bone alkaline phosphatase OR bone-specific alkaline phosphatase) OR (intact AND (pinp OR p1np OR procollagen 1 propeptide OR procollagen i propeptide))) AND (chronic kidney disease OR ckd OR end-stage kidney disease OR end-stage renal disease OR esrd OR eskd OR chronic renal failure OR crf OR kidney failure OR hemodialysis OR dialysis)) AND (pamidron* OR alendron* OR risedron* OR clodon* OR zoledron* OR ibandron* OR denosumab OR teriparatide OR romosozumab OR abaloparatide) [tiab] NOT review. Filter: English language. |
| Pediatric considerations | (Bone turnover markers) OR (bone formation markers) OR (bone alkaline phosphatase OR bAP) OR (alkaline phosphatase OR ALP) OR osteocalcin OR (procollagen I N-propeptide) or P1NP or PINP OR (bone resorption markers) OR (serum C-telopeptides of type I collagen) OR CTX OR (tartrate-resistant acid phosphatase type 5 b) OR TRACP5b OR TRAP5b or tartrate OR (tartrate-resistant) AND acid phosphatase OR urinary N-telopeptides of type I collagen OR NTX OR (parathyroid hormone OR PTH) AND (chronic kidney disease OR dialysis OR hemodialysis OR chronic renal insufficiency OR kidney failure) AND (paediatrics or children).  Filters: From 2000 up to date. Only English language. Humans. Children from birth-18 years  All studies including cohort, registry, cross-sectional observational studies, with retrospective studies limited to those with more than 20 children were included. In addition to the PubMed search, a manual review of the reference lists from the included studies and related reviews was conducted. |

Figure S1 Results from the Delphi survey on suggested clinical practice points of the consensus


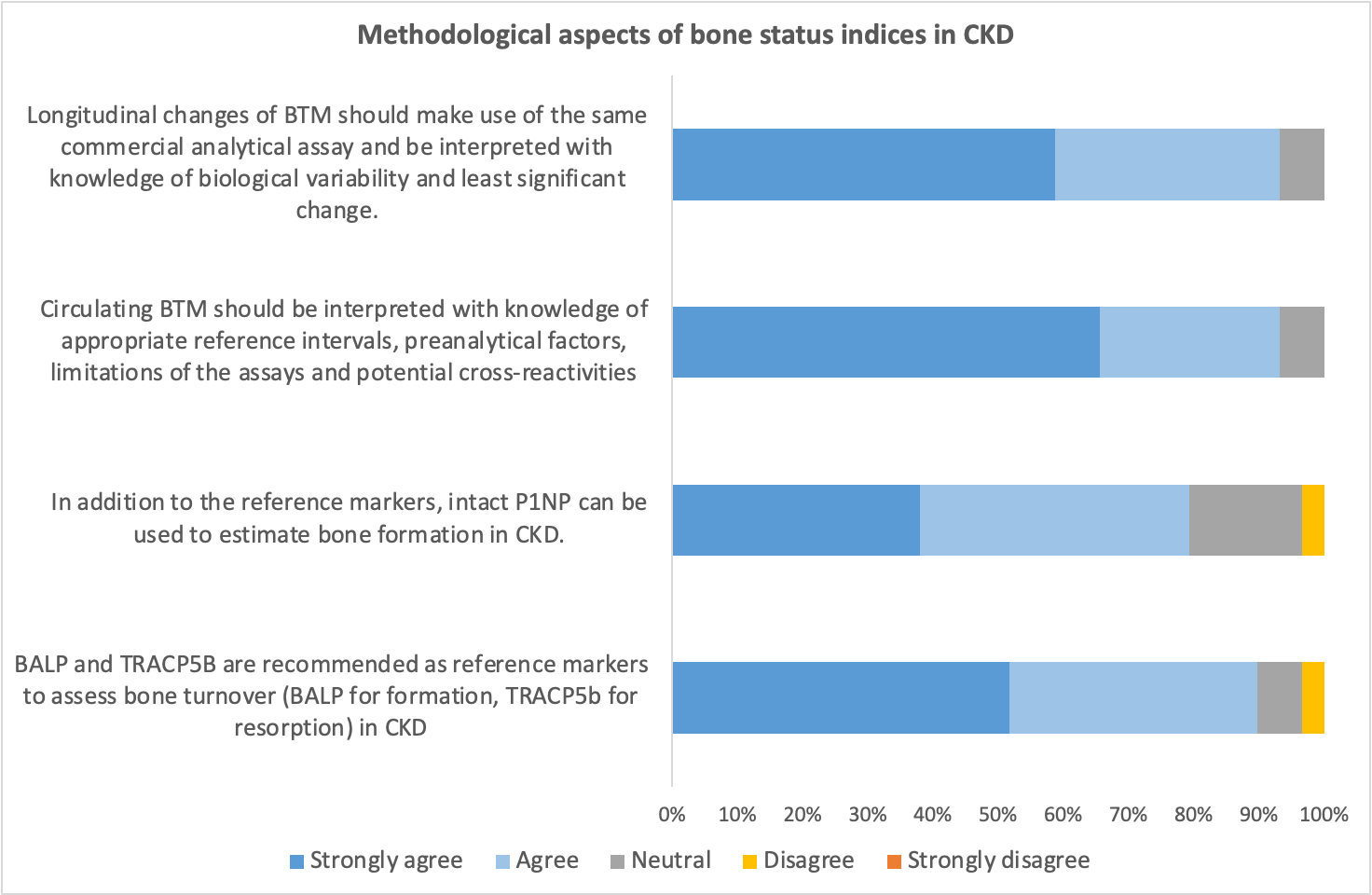


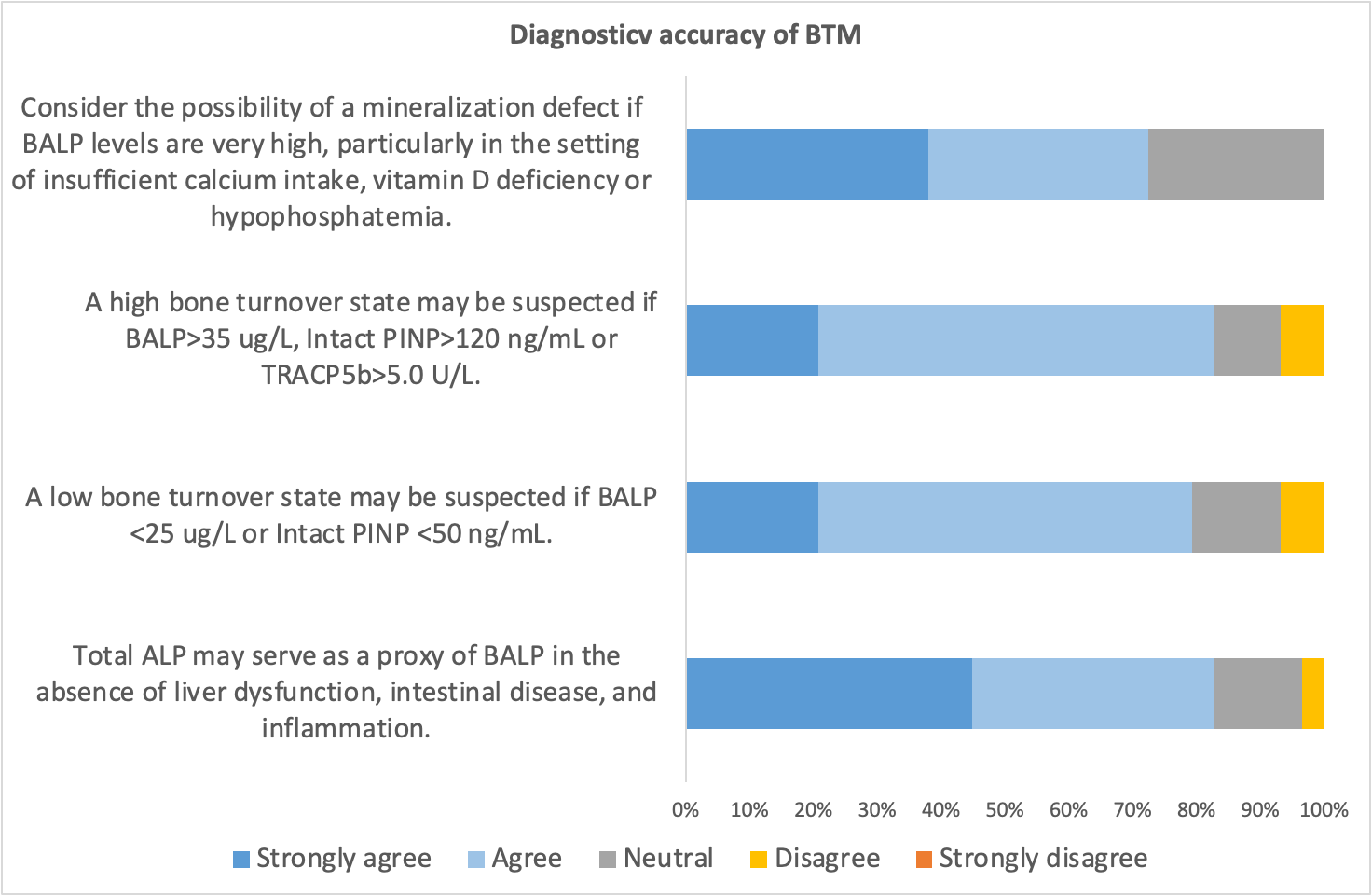


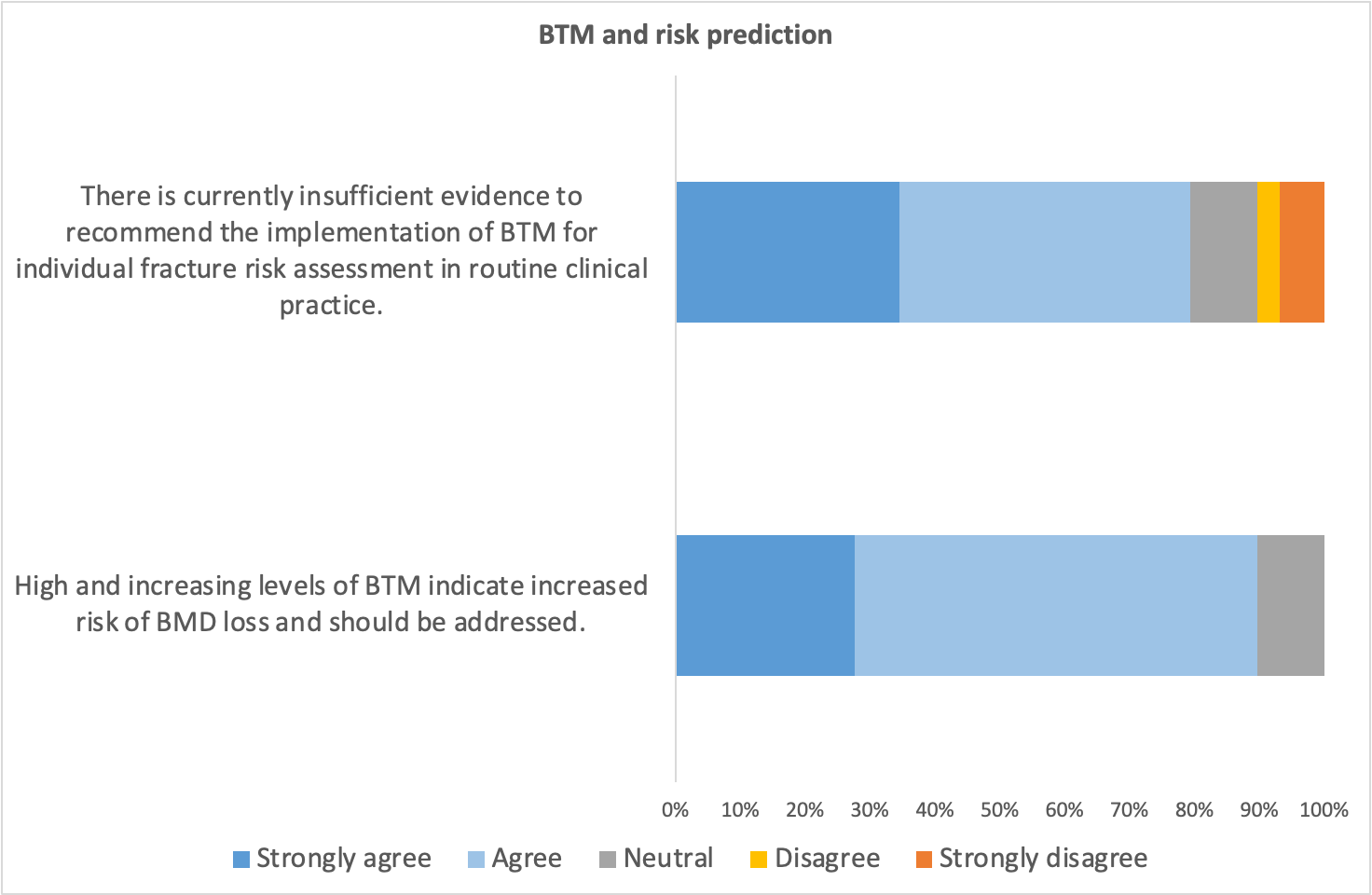


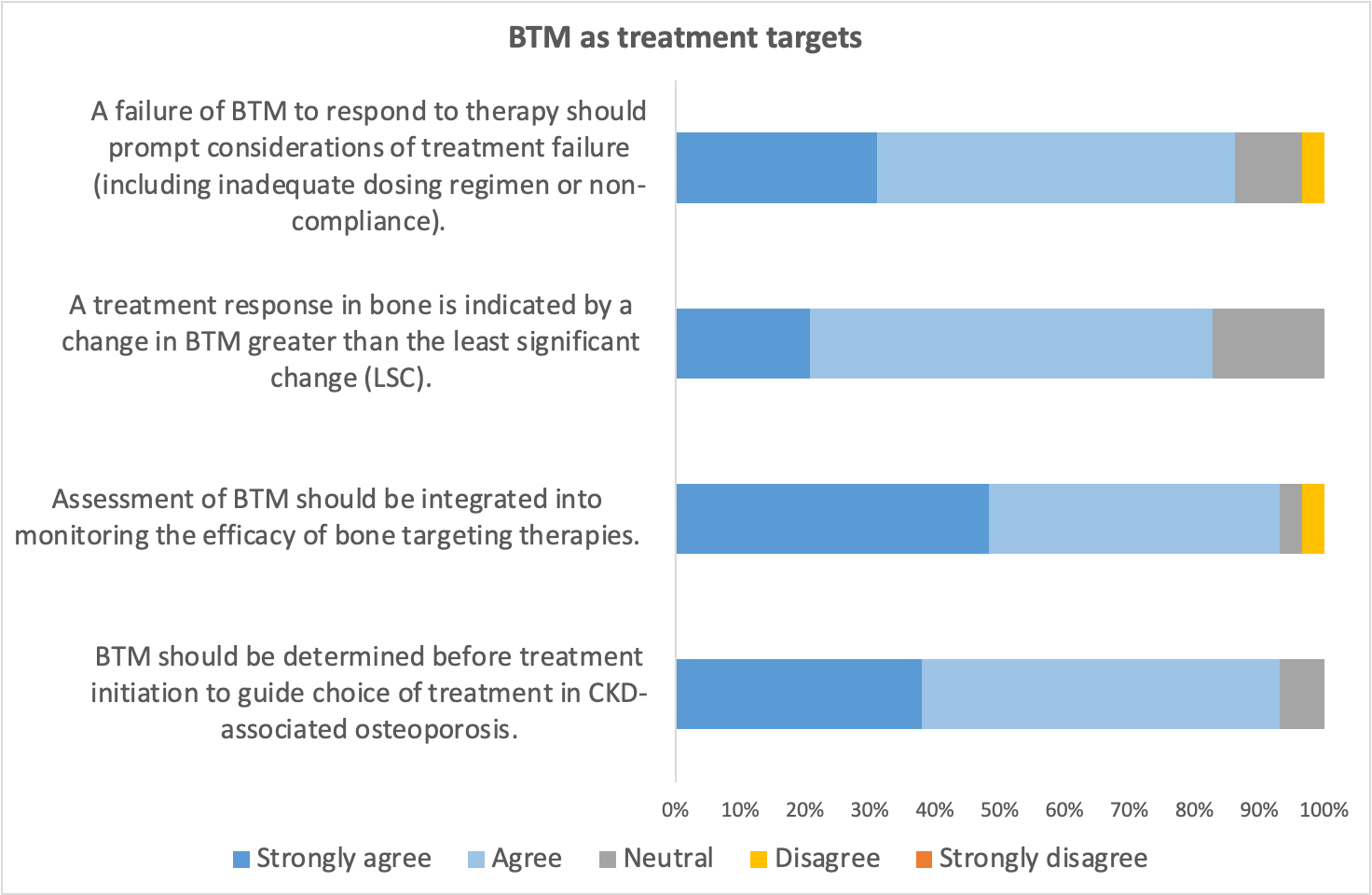


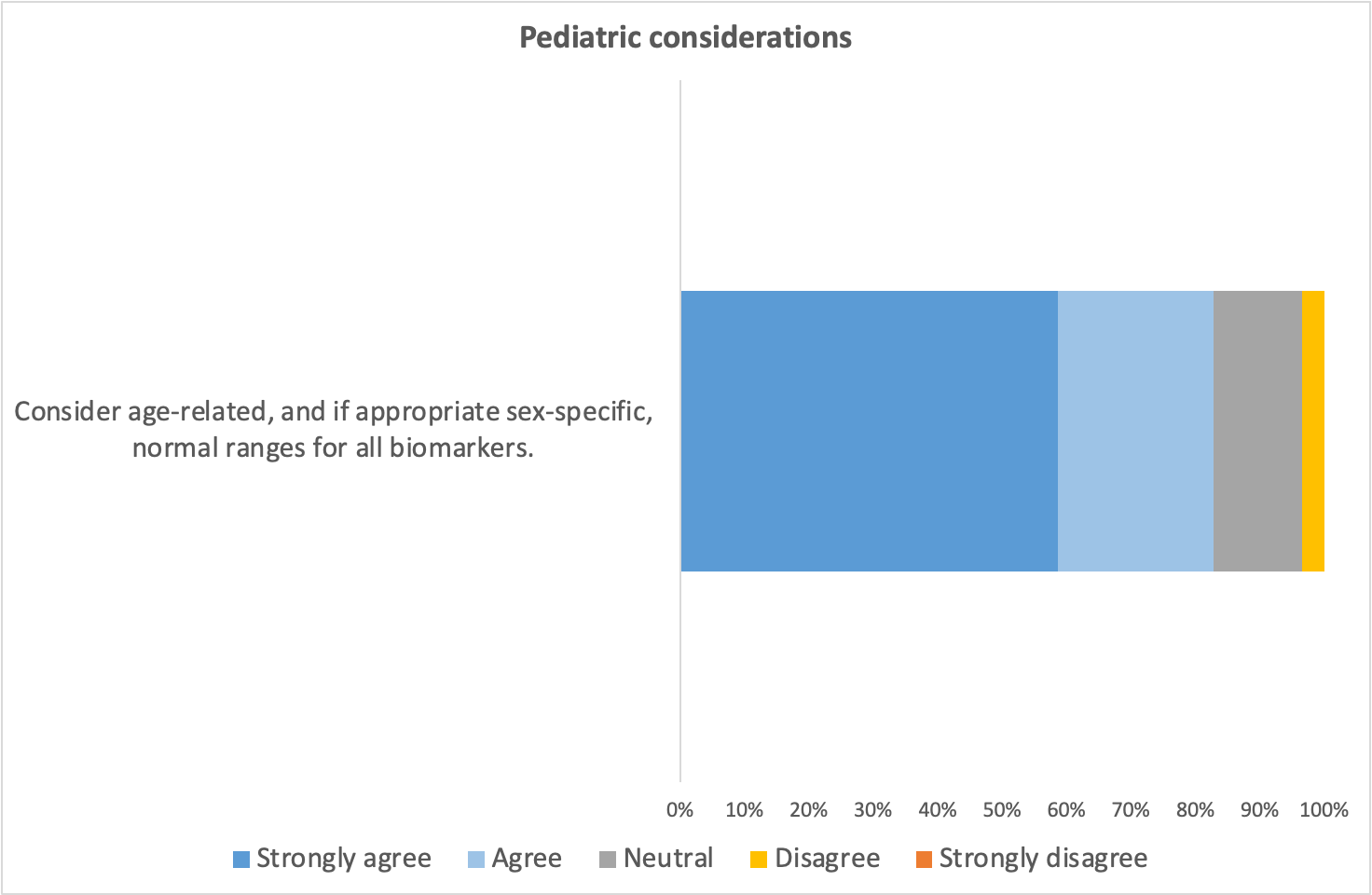


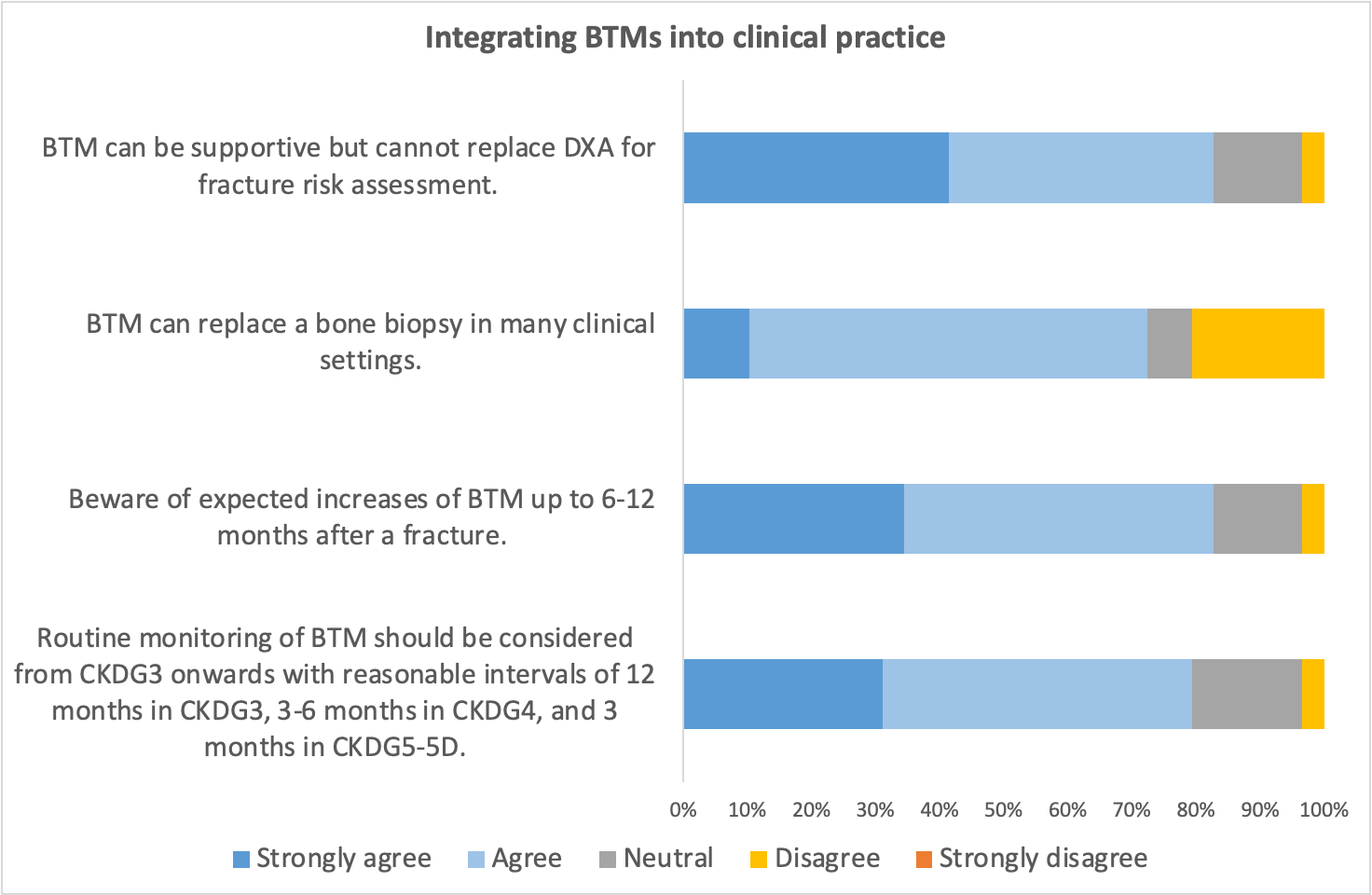

Supplement: gfag033_Supplemental_File [file gfag033_supplemental_file.docx]
